# Are physicians on the same page about do-not-resuscitate? To examine individual physicians’ influence on do-not-resuscitate decision-making: a retrospective and observational study

**DOI:** 10.1186/s12910-019-0429-z

**Published:** 2019-12-04

**Authors:** Yen-Yuan Chen, Melany Su, Shu-Chien Huang, Tzong-Shinn Chu, Ming-Tsan Lin, Yu-Chun Chiu, Kuan-Han Lin

**Affiliations:** 1 Department of Medical Education, Graduate Institute of Medical Education & Bioethics, National Taiwan University College of Medicine, National Taiwan University Hospital, #1, Rd. Ren-Ai sec. 1, Chong-Cheng District, Taipei, 10051 Taiwan; 2 New York University School of Medicine, #550 1st Avenue, New York, NY 10016 USA; 3 Department of Surgery, National Taiwan University Hospital, #7 Rd. Chong-Shan S, Taipei, 10002 Taiwan; 4 Graduate Institute of Medical Education & Bioethics, National Taiwan University College of Medicine, #1 Rd. Ren-Ai sec. 1, Chong-Cheng District, Taipei, 10051 Taiwan; 5 Department of Surgery, National Taiwan University College of Medicine, #1, Rd. Ren-Ai sec. 1, Chong-Cheng District, Taipei, 10051 Taiwan; 6 Department of Medical Education, National Taiwan University Hospital, #7, Rd. Chong-Shan S., Chong-Cheng District, Taipei, 10002 Taiwan; 7 Department of Healthcare Administration, Asia University, #500, Lioufeng Rd., Wufeng, Taichung, 41354 Taiwan

**Keywords:** Do-not-resuscitate, Intensive care, Decision-making

## Abstract

**Background:**

Individual physicians and physician-associated factors may influence patients’/surrogates’ autonomous decision-making, thus influencing the practice of do-not-resuscitate (DNR) orders. The objective of this study was to examine the influence of individual attending physicians on signing a DNR order.

**Methods:**

This study was conducted in closed model, surgical intensive care units in a university-affiliated teaching hospital located in Northern Taiwan. The medical records of patients, admitted to the surgical intensive care units for the first time between June 1, 2011 and December 31, 2013 were reviewed and data collected. We used Kaplan–Meier survival curves with log-rank test and multivariate Cox proportional hazards models to compare the time from surgical intensive care unit admission to do-not-resuscitate orders written for patients for each individual physician. The outcome variable was the time from surgical ICU admission to signing a DNR order.

**Results:**

We found that each individual attending physician’s likelihood of signing do-not-resuscitate orders for their patients was significantly different from each other. Some attending physicians were more likely to write do-not-resuscitate orders for their patients, and other attending physicians were less likely to do so.

**Conclusion:**

Our study reported that individual attending physicians had influence on patients’/surrogates’ do-not-resuscitate decision-making. Future studies may be focused on examining the reasons associated with the difference of each individual physician in the likelihood of signing a do-not-resuscitate order.

## Background

The development of intensive care units (ICUs) has introduced treatments and interventions that were previously unavailable at the end of life [1]. While end-of-life care (EOLC) has become increasingly aggressive over the last decade [2–4], studies have challenged the appropriateness of aggressive EOLC in certain circumstances. For example, higher intensity of care is not correlated with better clinical outcomes, higher family satisfaction, and a lower mortality rate [5–7]. Decisions to withhold or withdraw life-supporting treatments (LSTs) are respected when physicians deem such LSTs to no longer be beneficial to the patient, or when patients/their surrogate decision-makers, usually the patients’ family members, decline such LSTs [8]. Thus, while medical technology makes LSTs possible, there is still the opportunity to refuse such treatments and interventions, which serves to protect patient autonomy.

One form of EOLC that has received substantial legislative attention is Do-Not-Resuscitate (DNR) orders, the instruction for medical professionals not to attempt resuscitation on a patient when experiencing cardiac or respiratory arrest. In the United States, beginning in the 1970s, a series of policies and legislations were issued to address the lack of a structured decision-making process regarding cardiopulmonary resuscitation (CPR). In 1974, the American Heart Association approved the clinical use of CPR. It also suggested that an order not to resuscitate is appropriate for patients with irreversible clinical conditions, and should be documented in progress notes and communicated to hospital staff [9, 10]. The first law at the state level that specifically addressed DNR orders was the New York State Do-Not-Resuscitate Law of 1988 [11]. Partly for clearly indicating medical care provided to DNR patients, the State of Ohio established a Do-Not-Resuscitate Law in 1998, indicating two distinct protocols of DNR orders [12, 13]. In addition, Congress passed the Patient Self-Determination Act in 1990, which requires healthcare institutions to ask patients, upon admission, if they have prepared an advance directive, if they would like to place a copy in their records, and if they would like information about completing an advance directive [1].

Although laws may promote the practice of DNR orders when appropriate, DNR orders often fail to fulfill their intended purpose to promote patient self-determination and prevent non-beneficial medical interventions and treatments [14]. These unintended results may reflect the influence of external factors on the process of EOLC decision-making, including factors associated with the physician [15]. For example, physician specialty has a greater influence on the frequency and timing of DNR orders than does the presence of an advanced directive [16]. Results from a structured and scenario-based questionnaire distributed in several different Asian countries suggest that the likelihood that a physician will issue DNR orders is associated with whether they themselves had religious beliefs or were agnostics [17]. Lin et al. reported that the patients with the religious background of Buddhism/Daoism were less likely to consent to a DNR order [18]. By influencing the practice of DNR orders, these physician-associated factors may undermine patients’/surrogate decision-makers’ autonomy.

In Taiwan, DNR orders have been used in clinical medicine for several decades. It is mandatory that a physician must have the consent of the patient/surrogate decision-maker for issuing a DNR order. Although it has been emphasized that a DNR order only limits the initiation of CPR [19–21], it is never clear in Taiwan whether a DNR order limits only the use of CPR or whether in includes the use of other LSTs. After a DNR consent form is signed, the DNR order will always be in effect until a renunciation is documented.

Considering these findings, we conducted this study to examine the influence that attending physicians in surgical ICUs have on their patients’ DNR orders. We aimed to determine whether the likelihood of signing a DNR order for patients varies among individual physicians in actual clinical practice.

## Methods

### Setting

This study was conducted in closed model, surgical ICUs in a university-affiliated teaching hospital located in Northern Taiwan. There were 2040 beds available for inpatient care. Among them, 78 beds were in surgical ICUs. There were seven surgical ICUs with the total number of beds in each surgical ICU ranged from 9 to 13 during the data collection period. The medical services for caring for patients were shared by a team of physicians comprised of one attending physician and one or two house officers. The attending physician was responsible for all medical care decisions, including signing a DNR order.

### Study design

This is a retrospective and observational study. We included the patients aged 20 years or older, who were admitted to the surgical ICUs with a Therapeutic Intervention Scoring System (TISS) score, and who were cared for by only one attending physician with a surgical specialty during their stay in the surgical ICUs. Since a small number of participants may result in unreliable and unstable statistics, we also excluded the patients whose attending physicians cared for fewer than an absolute minimum of 10 patients [22]. The medical records of all patients who were admitted to the surgical ICUs for the first time between June 1, 2011 and December 31, 2013 were reviewed. The patients with a TISS score were mainly admitted to two of the surgical ICUs due to cardiothoracic illnesses. This study was approved by the Research Ethics Committee of the National Taiwan University Hospital (20140308RINC).

### Data collection

We collected the following variables: age, gender, religion, education, working status, marital status, residence, TISS score upon surgical ICU admission, length of surgical ICU stay, surgical ICU admission diagnosis, the status of DNR, date of DNR, the time from surgical ICU admission to signing a DNR order, and individual attending physicians. Time from surgical ICU admission to the signing of a DNR order was calculated. The outcome variable was the time from surgical ICU admission to signing a DNR order.

The TISS scoring system developed by Cullen et al. in 1974 is a set of 76 therapeutic tasks performed in ICUs. Higher TISS scores indicate a more severe illness and require a higher number of therapeutic interventions [23]. The surgical ICU admission diagnosis was collapsed to only four categories: (1) non-operative, cardiac failure/insufficiency; (2) non-operative, others; (3) post-operative, major surgery; and (4) post-operative, others.

### Statistical analysis

Patients eligible for this study were classified into two groups: (1) DNR patients; and (2) Non-DNR patients. We examined the differences in the independent variables between DNR patients and Non-DNR patients using Student’s t-test or Chi-square test depending on the scale of the independent variable. We also conducted Analysis of Variance (ANOVA) or Chi-square test to compare the differences in the personal characteristics of the patients cared for by each attending physician.

We used Kaplan–Meier survival curves to compare the time from surgical ICU admission to a physician’s signing a DNR order for patients. A physician’s signing a DNR order was considered “event”, and surgical ICU discharge was considered “censored” in this survival analysis. Differences in the Kaplan-Meier curves for the attending physicians were tested using log-rank tests.

We established a multivariate Cox proportional hazards model for examining the influence of attending physicians on their patients’/surrogate decision-makers’ DNR decision-making, using Physician 11 as the reference group, whose patient number was the median number among the patient number of the total 11 physicians. We compared each individual physician’s likelihood of signing a DNR order for a patient by comparing each individual attending physician’s time from his patient’s ICU admission to signing a DNR order for the patient. We created dummy variables for representing the attending physicians. A *p* value of less than or equal to .05 was considered statistically significant. All statistical analyses were conducted using SAS 9.2 (SAS Institute Inc., Cary, NC, USA).

## Results

### Patient characteristics

**Fig. 1:**
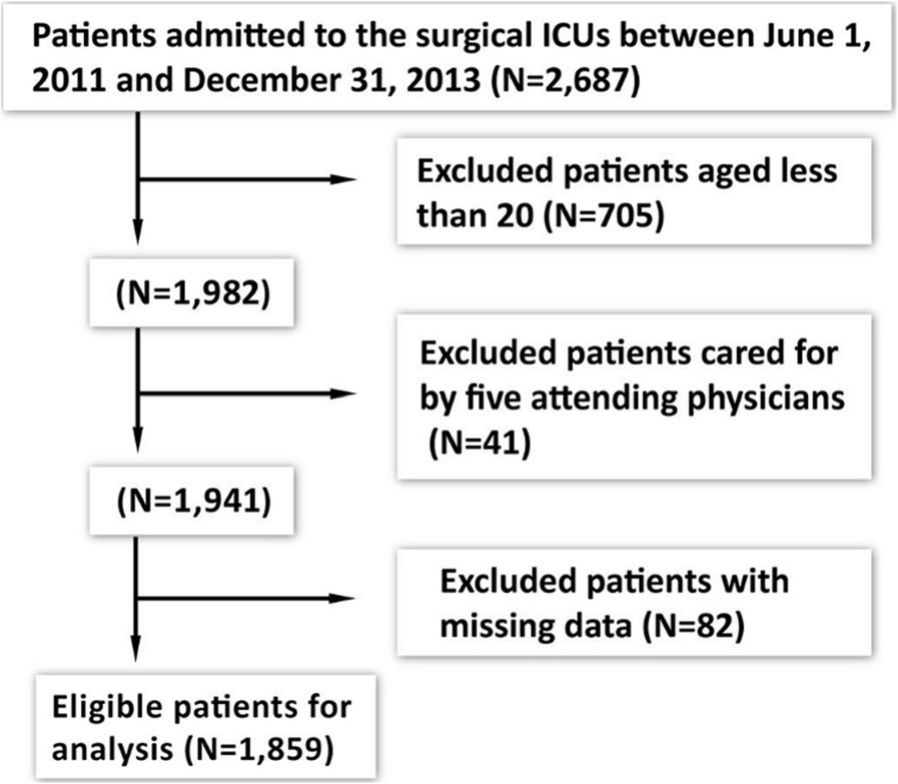
Study subjects enrollment

During the data collection period, 1982 patients (cared for by 16 attending physicians) were at the age of 20 years or older, admitted to the surgical ICUs with a TISS score, and were cared for by only one attending physician during their surgical ICU stay. A total of 41 patients (2.07%) cared for by one of the five attending physicians who cared for fewer than 10 patients during data collection period were excluded from this study. The patients cared for by the other 11 attending physicians were included. We also excluded 82 (4.14%) patients who had any missing data in any of the variables collected in this study. (Fig. 1).

A total of 1859 patients, with a mean age of 61.82 years, were eligible for this study. A total of 1254 patients (67.46%) were male. 45.62% of the 1859 patients reported that they were Buddhists/Daoists. Of the 1859 patients, 30.07% had educational years of 12 or longer, and most patients were married (76.98%). 62.18% of patients were not working fulltime, and only 5.27% were from rural areas. The mean TISS score of the 1859 patients was 32.03 (±10.78). Approximately half of the patients had the admission diagnosis of “non-operative, cardiac failure/insufficiency”. The average length of stay in surgical ICUs and in the hospital was 6.43 (±13.25) days and 24.5 (±28.74) days, respectively (Appendix 1).

### DNR and non-DNR patients

When compared to the Non-DNR patients, DNR patients were less likely to work fulltime (*p* = .01), admitted to the surgical ICUs with more severe clinical illness (*p* < .01), more likely to have the admission diagnosis of “non-operative, cardiac failure/insufficiency” (*p* < .01), and more likely to have a longer length of stay in surgical ICUs (*p* < .01) and in the hospital (*p* < .01). (Appendix 1).

### Personal characteristics stratified by attending physicians

Personal characteristics as stratified by the 11 attending physicians, i.e. gender (χ^2^ = 21.08, *p* = .02), age (F = 10.33, *p* < .01), education (χ^2^ = 32.17, *p* = .04), marital status (χ^2^ = 33.05, *p* < .01), admission diagnosis (χ^2^ = 486.32, *p* < .01), and TISS (F = 22.08, *p* < .01), were significantly different. (Table 1).

**Table 1:** Demographic and clinical characteristics of the patients care for by each individual attending physician

	P1 (*n* = 13)	P2 (*n* = 30)	P3 (*n* = 39)	P4 (*n* = 47)	P5 (*n* = 124)	P6 (*n* = 498)	P7 (*n* = 153)	P8 (*n* = 218)	P9 (*n* = 260)	P10 (*n* = 352)	P11 (*n* = 125)
Age	44.80~76.50	20.20~76.30	41.20~85.00	28.30~94.10	24.30~92.00	20.10~95.00	21.00~84.70	24.20~91.70	20.30~91.10	21.00~92.40	23.70~95.40
	57.30	52.90	68.00	69.80	71.10	64.75	58.20	60.60	59.50	62.10	63.10
	60.80 ± 10.54	52.46 ± 14.38	65.70 ± 12.06	68.77 ± 15.94	66.25 ± 17.29	64.65 ± 12.20	55.86 ± 16.39	60.81 ± 14.55	57.61 ± 17.85	61.88 ± 13.91	62.40 ± 14.20
Gender											
Male	11 (84.6%)	18 (60.00%)	25 (64.10%)	29 (61.70%)	97 (78.20%)	332 (66.70%)	100 (65.40%)	156 (71.60%)	157 (60.40%)	250 (71.00%)	79 (63.20%)
Female	2 (15.4%)	12 (40.00%)	14 (35.90%)	18 (38.30%)	27 (21.80%)	166 (33.30%)	53 (34.60%)	62 (28.40%)	103 (39.60%)	102 (29.00%)	46 (36.80%)
Religion											
BD	4 (30.80%)	12 (40.00%)	18 (46.20%)	24 (51.10%)	60 (48.40%)	240 (48.20%)	62 (40.50%)	90 (41.30%)	113 (43.50%)	171 (48.60%)	54 (43.20%)
CC	0	1 (3.30%)	2 (5.1%)	1 (2.10%)	6 (4.80%)	31 (6.20%)	8 (5.20%)	19 (8.70%)	7 (2.70%)	24 (6.80%)	9 (7.20%)
Others	9 (69.20%)	17 (56.70%)	19 (48.70%)	22 (46.80%)	58 (46.80%)	227 (45.60%)	83 (54.20%)	109 (50.00%)	140 (53.80%)	157 (44.60%)	62 (49.60%)
Education by year											
> 12	2 (15.40%)	8 (26.70%)	7 (17.90%)	12 (25.50%)	32 (25.80%)	170 (34.10%)	31 (20.30%)	67 (30.70%)	82 (31.50%)	106 (30.10%)	42 (33.60%)
1–12	11 (84.60%)	21 (70.00%)	28 (71.80%)	26 (55.30%)	79 (63.70%)	279 (56.00%)	113 (73.90%)	130 (59.60%)	149 (57.30%)	213 (60.50%)	72 (57.60%)
0	0	1 (3.30%)	4 (10.30%)	9 (19.10%)	13 (10.50%)	49 (9.80%)	9 (5.90%)	21 (9.60%)	29 (11.2%)	33 (9.40%)	11 (8.80%)
Marital Status											
Married	12 (92.30%)	19 (63.30%)	28 (71.80%)	28 (59.60%)	86 (69.40%)	410 (82.30%)	108 (70.60%)	170 (78.00%)	191 (73.50%)	282 (80.10%)	97 (77.60%)
Others	1 (7.70%)	11 (36.70%)	11 (28.20%)	19 (40.40%)	38 (30.60%)	88 (17.70%)	45 (29.40%)	48 (22.00%)	69 (26.50%)	70 (19.90%)	28 (22.40%)
Working Fulltime											
No	8 (61.50%)	18 (60.00%)	23 (59.00%)	37 (78.70%)	82 (66.10%)	318 (63.90%)	92 (60.10%)	120 (55.00%)	156 (60.00%)	229 (65.10%)	72 (58.40%)
Yes	5 (38.50%)	12 (40.00%)	16 (41.00%)	10 (21.30%)	42 (33.90%)	180 (36.10%)	61 (39.90%)	98 (45.00%)	104 (40.00%)	123 (34.90%)	52 (41.60%)
Residence											
Rural	0	2 (6.70%)	1 (2.60%)	1 (2.10%)	7 (5.60%)	16 (3.20%)	15 (9.80%)	9 (4.10%)	16 (6.20%)	24 (6.80%)	7 (5.60%)
Urban	13 (100.00%)	28 (93.30%)	38 (97.40%)	46 (97.90%)	117 (94.40%)	482 (96.80%)	138 (90.20%)	209 (95.90%)	244 (93.80%)	328 (93.20%)	118 (94.40%)
Admission Diagnosis											
NO, CF/I	6 (46.20%)	10 (33.30%)	29 (74.40%)	10 (21.30%)	12 (9.70%)	273 (54.80%)	100 (65.40%)	116 (53.20%)	111 (42.70%)	172 (48.90%)	53 (42.40%)
NO, others	0	10 (33.30%)	0	4 (8.50%)	10 (8.10%)	3 (0.60%)	20 (13.10%)	6 (2.80%)	41 (15.80%)	8 (2.30%)	1 (0.80%)
O, MS	3 (23.10%)	6 (20.00%)	8 (20.50%)	12 (25.50%)	20 (16.10%)	169 (33.90%)	22 (14.40%)	63 (28.90%)	78 (30.00%)	102 (29.00%)	59 (47.20%)
O, others	4 (30.80%)	4 (13.30%)	2 (5.10%)	21 (44.70%)	82 (66.10%)	53 (10.60%)	11 (7.20%)	33 (15.10%)	30 (11.50%)	70 (19.90%)	12 (9.60%)
TISS	16~48	11~63	15~77	10~53	6~67	7~65	7~62	7~67	2~83	6~67	7~62
	31.00	36.50	36.58	20.00	20.00	34.00	33.00	32.00	36.00	32.00	34.00
	29.46 ± 10.03	36.10 ± 11.78	37.23 ± 11.55	21.09 ± 8.43	23.06 ± 11.12	33.40 ± 8.20	32.69 ± 11.45	30.72 ± 11.06	35.61 ± 12.09	30.94 ± 9.84	34.41 ± 10.09
LOS by day											
SICU	0~27	1~369	0~18	0~43	0~31	0~105	0~74	0~70	0~132	0~87	0~53
	3	3	2	2	2	4	4	4	3	3	3
	6.92 ± 9.09	15.97 ± 66.73	3.56 ± 4.33	5.04 ± 7.78	3.58 ± 5.40	6.18 ± 10.25	5.79 ± 8.31	6.47 ± 8.90	7.37 ± 13.19	7.13 ± 11.88	6.02 ± 8.98
Hospital	6~94	1~375	6~76	1~533	0~124	1~207	0~177	0~209	0~185	2~231	0~318
	19	15	16	11	11	15	16	18	18	18	16
	30.00 ± 28.31	26.47 ± 66.35	21.46 ± 15.56	35.32 ± 11.95	20.65 ± 24.73	21.62 ± 23.53	20.60 ± 20.65	26.58 ± 24.95	26.02 ± 24.14	27.32 ± 25.85	25.66 ± 31.64

Abbreviation List: *P* Physician, *BD* Buddhist/Daoist, *CC* Christian/Catholics, *Marital* Marital Status, *NO, CF/I* Non-operative, cardiac failure/insufficiency, *O, MS* Operative, major surgery, *TISS* Therapeutic Intervention Scoring System, *LOS* length of stay, *SICU* surgical intensive care unit

Continuous variables such as Age, Education by Year, TISS, and Length of Stay by Day are reported as “minimal number ~ maximal number, median, mean ± standard deviation”

Categorical variables are reported as “total number (percentage)”

### The differences of individual physicians to write a DNR order

**Fig. 2:**
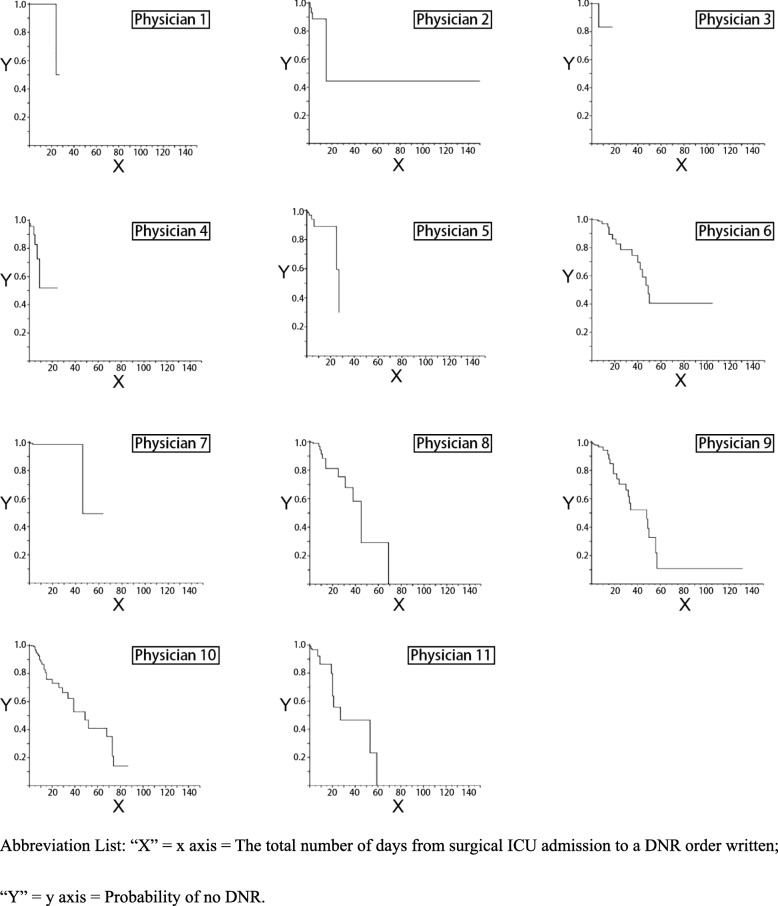
The probability of signing do-not-resuscitate orders for each individual attending physician

In Fig. 2, Kaplan-Meier survival curves demonstrate the length of time after surgical ICU admission that patients remained no DNR order. Log-rank tests showed that the probability of remaining no DNR order after surgical ICU admission was significantly different for the 11 attending physicians (log-rank chi-square 31.40, *p* < .01).

Since patients characteristics as stratified by each attending physician varied, multivariate Cox proportional hazards regression analysis was used to adjust for the combined effect of confounding variables (i.e. gender, age, religion, education, marital status, working status, residence, admission diagnosis, and TISS) [24]. The hazard ratios for each individual physician ranged from 0.26 to 3.82 after controlling for other confounding variables, with the ratio of the largest to the smallest hazard ratios equaling 14.7 (Physician 4 = 3.82 and Physician 7 = 0.26). Compared to Physician 11, Physician 4 was more likely to write a DNR order (hazard ratio = 3.82, *p* < .01), but Physician 6 (hazard ratio = 0.34, *p* < .01) and Physician 7 (hazard ratio = 0.26, *p* = .04) were less likely to write a DNR order for their patients. In addition, the patient’s diagnosis of “Non-operative, cardiac failure/insufficiency” upon surgical ICU admission (*p* < .01) and severity of clinical illness (*p* < .01) were associated with signing a DNR order. (Table 2) (Appendix 2).

**Table 2:** Cox proportional hazards models for signing a DNR order

	Crude Hazard Ratio (95% CI)	*P* value	Adjusted Hazard Ratio (95% CI)	*P* value
Gender				
Male	0.68 (0.52–1.12)	0.18	0.90 (0.56–1.42)	0.64
Female	1.0		1.0	–
Age, year	1.01 (1.00–1.02)	0.10	1.01 (1.00–1.03)	0.10
Religion				
Others	0.56 (0.27–1.18)	0.13	0.59 (0.27–1.28)	0.18
Buddhist/Daoist	0.58 (0.27–1.23)	0.16	0.60 (0.27–1.31)	0.20
Christian/Catholic	1.0		1.0	–
Education, year				
> 12	0.62 (0.33–1.17)	0.14	0.81 (0.37–1.79)	0.60
1–12	0.60 (0.33–1.07)	0.08	0.81 (0.42–1.56)	0.52
0	1.0		1.0	–
Marital Status				
Married	0.85 (0.57–1.25)	0.40	0.88 (0.56–1.37)	0.56
Others	1.0		1.0	–
Working Fulltime				
Yes	0.77 (0.51–1.15)	0.20	0.81 (0.49–1.34)	0.41
No	1.0		1.0	
Residence				
Rural area	1.77 (0.86–3.65)	0.12	1.76 (0.83–3.76)	0.14
Urban area	1.0		1.0	
Admission Diagnosis				
Non-operative ^a^, cardiac failure/insufficiency	1.54 (0.93–2.55)	0.09	2.49 (1.37–4.53)	< 0.01
Non-operative, others	1.70 (0.64–4.58)	0.30	2.45 (0.84–7.16)	0.10
Post-operative ^b^, major surgery	0.91 (0.50–1.80)	0.79	1.19 (0.56–2.53)	0.66
Post-operative others	1.0		1.0	–
TISS	1.02 (1.00–1.03)	0.04	1.02 (1.00–1.04)	< 0.01
Individual Physicians				
Physician 1	0.75 (0.10–5.77)	0.78	0.71 (0.09–5.59)	0.75
Physician 2	1.02 (0.33–3.21)	0.97	1.42 (0.42–4.77)	0.57
Physician 3	0.62 (0.08–4.77)	0.65	0.47 (0.06–3.70)	0.47
Physician 4	2.64 (1.04–6.70)	0.04	3.82 (1.40–10.45)	< 0.01
Physician 5	1.22 (0.48–3.08)	0.68	2.09 (0.77–5.68)	0.15
Physician 6	0.36 (0.17–0.74)	< 0.01	0.34 (0.16–0.71)	< 0.01
Physician 7	0.28 (0.08–0.99)	0.05	0.26 (0.07–0.95)	0.04
Physician 8	0.60 (0.28–1.31)	0.20	0.53 (0.24–1.19)	0.12
Physician 9	0.72 (0.37–1.43)	0.35	0.66 (0.33–1.34)	0.25
Physician 10	0.65 (0.34–1.25)	0.20	0.71 (0.35–1.46)	0.36
Physician 11	1.0		1.0	–

Abbreviation List: *DNR* do-not-resuscitate, *CI* confidence interval, *TISS* Therapeutic Intervention Scoring System, *ICU* intensive care unit

^a^ “Non-operative” implies that patients: (1) have undergone cardiothoracic surgical procedures before this admission, and were admitted due to clinical illnesses associated with prior surgical procedures; (2) were scheduled to receive a cardiothoracic surgical procedure; or (3) currently being supported by extracorporeal membrane oxygenation (ECMO)

^b^ “Post-operative” implies that patients have undergone cardiothoracic surgical procedures in this admission

## Discussion

### Main outcomes

This study showed that, after controlling for other confounding variables, attending physicians influenced the DNR decision made by their patients and the patients’ surrogate decision-makers. Some physicians were more likely to write a DNR order for the patients, and others were less likely to do so. The patient’s admission diagnosis and severe clinical illness upon surgical ICU admission were associated with signing a DNR order.

### Discussion for do-not-resuscitate decision

Groselj et al. reported that the most frequent type of termination of LSTs that ICU physicians encounter is DNR, and 97% of the discussions about the limitations of LSTs in ICUs are initiated by physicians [25]. In addition, physicians can influence DNR decisions by the timing in which they mention them, i.e. early or late in disease course, and by how and what they say. For example, 65% of physicians caring for cancer patients tend to discuss EOL decision-making when an asymptomatic cancer patient has four to six months to live, whereas 15% of physicians prefer to discuss the topic only if the patients/surrogate decision-makers bring it up [26]. Hilden et al. also found that although most physicians feel capable of discussing EOL decision-making, they still feel that education for EOL decision-making is necessary [27]. These studies illustrate that physicians play a very important role in DNR decision-making discussions, and these studies also highlight the need for more extensive training related to DNR decision-making, as well as physician-patient/physician-surrogate communication.

### Physician characteristics and do-not-resuscitate

Several studies have reported that the physician’s specialty is associated with whether DNR orders were written. After reviewing study results from the Study to Understand Prognoses and Preferences for Outcomes and Risks of Treatments (SUPPORT), Covinsky et al. reported that the physician’s specialty was a strong determinant of signing a DNR order [28]. Kelly et al. asked the physicians with different subspecialties regarding their preference to recommend DNR to patients based on 20 vignettes. They found that the physicians from pulmonary/critical care medicine were more likely to recommend DNR to patients than those from cardiology, or from general internal medicine [29]. Morrell et al. conducted a chart review study to examine the association between physician specialty and DNR orders for the patients they cared for. They reported that physicians with a medical specialty or subspecialty were more likely to write a DNR order than those with a surgical specialty or surgical subspecialty [16].

To control for the influence of a different specialty on DNR decision-making, our study further narrowed down the results to the attending physicians with a surgical specialty in the surgical ICUs. We identified that, even in the same specialty, some physicians were more likely to write a DNR order for patients than other physicians. In addition to physicians’ specialty/subspecialty, other factors of the physicians may also influence the timing of their willingness to discuss DNR issues with patients/surrogate decision-makers, and write a DNR order for patients.

Among those factors, physicians’ interpretation of DNR may play a very important role regarding the timing of signing a DNR order. Although it has been highlighted that DNR only limits the initiation of CPR, not the medical care provided to patients before the initiation of CPR [19–21], healthcare professionals are still confused with the medical care provided to patients with DNR orders. Some of them may interpret DNR patients as eligible to receive aggressive interventions and treatments to extend life before cardiac or respiratory arrest occurs if those interventions and treatments are ethically appropriate [30, 31], however, other healthcare professionals may interpret DNR patients as eligible to receive only comfort care measures [32–34]. Therefore, if the attending physicians in our study considered medical care for their DNR patients in a manner similar to the previous interpretation, they might be more likely to discuss DNR issues with patients/surrogate decision-makers and write a DNR order for patients earlier compared to attending physicians who considered interventions for DNR patients in a manner similar to the latter interpretation. As a result, physicians’ interpretations regarding the medical care provided to patients after a DNR order is written are associated with the timing of their decisions-making to write a DNR order for patients.

Physician communication with patients/surrogate decision-makers, other things being equal, may influence the timing of DNR decision-making. Physicians’ abilities to communicate with patients/surrogate decision-makers about DNR decision-making may vary. Physicians who lack a good quality of communication with patients/surrogate decision-makers about DNR decision-making may not conduct a successful DNR discussion [35], and may see discussing with patients/surrogates about DNR decision-making as a low priority, thus delaying the timing of signing DNR orders. In comparison, other physicians may be good at discussing DNR decision-making and be more willing to communicate with patients/surrogate decision-makers, thus not delaying the timing of signing DNR orders. Accordingly, the physicians’ communication concerning DNR decision-making may influence the timing of issuing DNR orders.

Although physicians must have understood DNR to a certain degree given that issues related to DNR have been taught in medical schools and in continuing medical education, signing a DNR order for patients, in addition to DNR interpretation and communication, is still deeply ingrained in: (1) the different specialty or subspecialty training that physicians receive on the issues of DNR and communication with patients [16, 29, 36]; (2) physicians’ characteristics such as their level of medical training [37], religious background [18, 38, 39], and so on; and (3) medical and organizational practice [16, 29, 40]. To examine the relationship between each of the physicians’ individual personal characteristics, and between the physicians’ personal characteristics and signing a DNR order, more studies should be conducted using factor analysis and/or structural equation modeling with path analysis.

### Strengths and limitations

Compared with prior studies using questionnaires to measure physicians’ attitudes toward signing a DNR order, our study further examined the actual practice of DNR decision-making for patients cared for by each individual attending physician. In addition, this study was narrowed down to a single specialty by focusing on the attending physicians with surgical specialty. Nevertheless, there are some limitations in this study.

This is a single-center study conducted in a medical center located in northern Taiwan. Our study sample was older, had more males, and fewer Buddhists/Daoists than the population of Taiwan [41]. Therefore, the generalizability of the study results to other different healthcare institutions, or to other physicians with a different specialty should be carefully deliberated.

The second limitation is that some potential confounding variables might not be included in the multivariate Cox proportional hazards model. Although we tried to adjust for all available confounding variables in the dataset, there might have been other confounding variables that were not adjusted for in the model. For example, there might be some possibility that the DNR decision-making was also influenced by the house officer and nurse. The influence of other healthcare team members on DNR decision-making was not measured and adjusted for in the multivariate Cox proportional hazards model.

The third limitation is that the DNR order was not always consented to by the patient himself/herself. Some DNR orders were consented to by surrogate decision-makers, and others were consented to jointly by patients and surrogate decision-makers. Nevertheless, EOLC decision-making studies that did not control for decision-makers still had academic merits [42]. Accordingly, the concern that the DNR decision-maker was not controlled for in this study could be set aside.

The fourth limitation is that only the 11 attending physicians were included when examining the relationship between an individual attending physician and DNR decision-making. The five attending physicians who cared for a total of 41 patients during data collection were excluded because of the small number of patients they cared for compared to the 11 attending physicians. Nevertheless, the validity of this study was not hurt given that the excluded 41 patients accounted for only 2.21% of the 1859 study subjects. The study results are still convincing and has acceptable generalizability even though the five attending physicians were excluded from this study.

The fifth limitation is that the traditional factor analysis and/or structural equation modeling could not be used to test for physician characteristics associated with the likelihood of writing a DNR order. Factor analysis and structural equation modeling are assumed that the relationships between the factors and the observed variables are linear, in this study the model includes variables that are continuous and nominal, which is not suitable for performing these analyses.

## Conclusions

Our study reported that individual physicians had influence on the timing of signing a DNR order for patients. Some of them were more likely to write a DNR order for their patients than others were. Future studies may be focused on examining the reasons associated with the difference of each individual physician concerning the likelihood of signing a DNR order. Educational interventions may be executed to prevent misinterpretation by medical professionals regarding DNR orders and to facilitate the discussion between medical professionals/other healthcare team members and patients/surrogate decision-makers.

## Data Availability

The dataset for this study is available on reasonable request by qualified researchers to Drs. Yen-Yuan Chen and Kuan-Han Lin.

## Appendix 1

**Table 3:** Demographic and clinical characteristics of DNR and Non-DNR patients

	Total (*n* = 1859)	DNR (*n* = 119)	Non-DNR (*n* = 1740)	*P* value
	N (%)	N (%)	N (%)	
Age, year (Mean ± SD)	61.82 ± 15.03	64.00 ± 15.60	61.67 ± 14.98	0.10
Gender				0.80
Female	605 (32.54)	40 (33.61)	565 (32.47)	
Male	1254 (67.46)	79 (66.39)	1175 (67.53)	
Religion				0.10
Buddhist/Daoist	848 (45.62)	43 (36.13)	805 (46.26)	
Christian/Catholics	108 (5.81)	8 (6.72)	100 (5.75)	
Others	903 (48.58)	68 (57.14)	835 (47.99)	
Education, year				0.60
> 12	559 (30.07)	32 (26.89)	527 (30.29)	
1–12	1121 (60.30)	73 (61.35)	1048 (60.23)	
0	179 (9.63)	14 (11.77)	165 (9.48)	
Marital Status				0.06
Married	1431 (76.98)	83 (69.75)	1348 (77.47)	
Others	428 (23.02)	36 (30.25)	392 (22.53)	
Working Fulltime				0.01
No	1156 (62.18)	86 (72.27)	1070 (61.49)	
Yes	703 (37.82)	33 (27.73)	670 (38.51)	
Residence				0.48
Rural area	98 (5.27)	8 (6.72)	90 (5.17)	
Urban area	1761 (94.73)	111(93.28)	1650 (94.83)	
Admission Diagnosis				< 0.01
Non-operative ^a^, cardiac failure/insufficiency	892 (47.98)	80 (67.23)	812 (46.67)	
Non-operative, others	103 (5.54)	5 (4.20)	98 (5.63)	
Post-operative ^b^, major surgery	542 (29.16)	15 (12.61)	527 (30.29)	
Post-operative, others	322 (17.32)	19 (16.97)	303 (17.41)	
TISS	32.03 ± 10.78	37.71 ± 13.72	31.65 ± 10.45	< 0.01
$$ \mathrm{Length}\ \mathrm{of}\ \mathrm{Stay},\mathrm{day}\ \left(\frac{\mathrm{Mean}\pm \mathrm{SD}}{\mathrm{Median}/\mathrm{IQR}}\right) $$				
Surgical ICU	6.43 ± 13.25	19.08 ± 20.62	5.56 ± 12.13	< 0.01
	3 / 4	12 / 19	3 / 3	
Hospital	24.50 ± 28.74	35.28 ± 56.53	23.76 ± 25.63	< 0.01
	16 / 13	20 / 40	16 / 12.75	

Abbreviation List: *DNR* do-not-resuscitate, *TISS* Therapeutic Intervention Scoring System, *ICU* intensive care unit, *SD* standard deviation, *IQR* interquartile range

^a^ “Non-operative” implies that the patients: (1) have undergone cardiothoracic surgical procedures before this admission, and were admitted due to clinical illnesses associated with prior surgical procedures; (2) were scheduled to receive a cardiothoracic surgical procedure; or (3) currently being supported by extracorporeal membrane oxygenation (ECMO)

^b^ “Post-operative” implies that patients have undergone cardiothoracic surgical procedures in this admission

## Appendix 2

**Table 4:** The results of multivariate Cox proportional hazards model by different reference physician

	Reference										
	P1	P 2	P 3	P 4	P 5	P 6	P 7	P 8	P 9	P 10	P 11
P 1	1.0	0.50 (0.05–4.90)	1.51 (0.09–25.02)	0.19 (0.02–1.63)	0.34 (0.04–2.92)	2.10 (0.27–16.13)	2.70 (0.28–16.49)	1.34 (0.17–10.36)	1.07 (0.14–8.12)	0.99 (0.13–7.51)	0.71 (0.09–5.59)
P 2	2.00 (0.20–19.63)	1.0	3.03 (0.32–28.78)	0.37 (0.10–1.41)	0.68 (0.17–2.66)	4.19 (1.29–13.66)	5.40 (1.13–25.80)	2.68 (0.77–9.33)	2.14 (0.67–6.80)	1.99 (0.53–6.34)	1.42 (0.42–4.77)
P 3	0.66 (0.04–10.94)	0.33 (0.04–3.14)	1.0	0.12 (0.01–1.08)	0.23 (0.03–1.96)	1.39 (0.18–10.73)	1.79 (0.18–17.69)	0.88 (0.11–7.01)	0.71 (0.09–5.44)	0.66 (0.09–5.12)	0.47 (0.06–3.70)
P 4	5.38 (0.61–47.26)	2.69 (0.71–10.21)	8.14 (0.93–71.71)	1.0	1.83 (0.62–5.41)	11.28 (4.24–30.00)	14.53 (3.51–60.23)	7.20 (2.53–20.51)	5.76 (2.20–15.03)	5.35 (2.09–13.72)	3.82 (1.40–10.45)
P 5	2.94 (0.34–25.25)	1.47 (0.38–5.74)	4.45 (0.51–38.85)	0.55 (0.19–1.61)	1.0	6.16 (2.32–16.38)	7.94 (1.94–32.50)	3.93 (1.43–10.85)	3.14 (1.21–8.20)	2.92 (1.15–7.41)	2.09 (0.77–5.68)
P 6	0.48 (0.06–3.68)	0.24 (0.07–0.78)	0.72 (0.09–5.59)	0.09 (0.03–0.24)	0.16 (0.06–0.43)	1.0	1.29 (0.37–4.46)	0.64 (0.30–1.35)	0.51 (0.27–0.98)	0.48 (0.25–0.89)	0.34 (0.16–0.71)
P 7	0.37 (0.04–3.64)	0.19 (0.04–0.88)	0.56 (0.06–5.55)	0.07 (0.02–0.29)	0.13 (0.03–0.52)	0.78 (0.22–2.69)	1.0	0.49 (0.14–1.77)	0.40 (0.12–1.35)	0.37 (0.11–1.23)	0.26 (0.07–0.95)
P 8	0.75 (0.10–5.79)	0.37 (0.11–1.30)	1.13 (0.14–8.96)	0.14 (0.05–3.40)	0.25 (0.09–0.70)	1.57 (0.74–3.31)	2.02 (0.57–7.21)	1.0	0.80 (0.39–1.65)	0.74 (0.36–1.51)	0.53 (0.24–1.19)
P 9	0.93 (0.12–7.10)	0.47 (0.15–1.48)	1.42 (0.18–10.88)	0.17 (0.07–0.45)	0.32 (0.12–0.83)	1.96 (1.02–3.77)	2.53 (0.74–8.58)	1.25 (0.61–2.58)	1.0	0.93 (0.52–1.68)	0.66 (0.33–1.34)
P 10	1.01 (0.13–7.60)	0.50 (0.16–1.60)	1.52 (0.20–11.82)	0.19 (0.07–0.48)	0.34 (0.14–0.87)	2.11 (1.12–3.96)	2.72 (0.81–9.09)	1.35 (0.66–2.72)	1.08 (0.60–1.94)	1.0	0.71 (0.35–1.46)
P 11	1.41 (0.18–11.10)	0.70 (0.21–2.36)	2.13 (0.27–16.82)	0.26 (0.10–0.72)	0.48 (0.18–1.31)	2.95 (1.40–6.23)	3.81 (1.06–13.72)	1.89 (0.84–4.22)	1.51 (0.75–3.05)	1.40 (0.69–2.87)	1.0

Abbreviation List: *P* Physician

The results were demonstrated by hazards ration (95% confidence interval)

## Abbreviations

EOLC: End-of-life care
LST: Life-supporting treatments
DNR: Do-not-resuscitate
CPR: Cardiopulmonary resuscitation
ICU: Intensive care unit
TISS: Therapeutic intervention scoring system
ECMO: Extracorporeal membrane oxygenation
